# The Indirect Effect of an Internet-Based Intervention on Third-Party Disability for Significant Others of Individuals with Tinnitus

**DOI:** 10.3390/audiolres14050068

**Published:** 2024-09-13

**Authors:** Eldré W. Beukes, Gerhard Andersson, Vinaya Manchaiah

**Affiliations:** 1 Vision and Hearing Sciences Research Group, School of Psychology and Sports Sciences, Anglia Ruskin University, Cambridge CB1 1PT, UK; 2 Virtual Hearing Lab, Collaborative Initiative between University of Pretoria and University of Colorado School of Medicine, Aurora, CO 80045, USA; vinaya.manchaiah@cuanschutz.edu; 3 Department of Behavioral Sciences and Learning, Linköping University, 582 25 Linköping, Sweden; gerhard.andersson@liu.se; 4 Department of Clinical Neuroscience, Division of Psychiatry, Karolinska Institute, 171 77 Stockholm, Sweden; 5 Department of Otolaryngology-Head and Neck Surgery, University of Colorado School of Medicine, Aurora, CO 80045, USA; 6 UCHealth Hearing and Balance, University of Colorado Hospital, Aurora, CO 80045, USA; 7 Department of Speech-Language Pathology and Audiology, University of Pretoria, Pretoria 0001, South Africa; 8 Department of Speech and Hearing, School of Allied Health Sciences, Manipal University, Manipal 576104, India

**Keywords:** significant others, third-party disability, tinnitus, tinnitus treatment, internet-intervention, tinnitus effects

## Abstract

Background: This study aimed to investigate whether Internet-based cognitive behavioural therapy intervention (ICBT) for individuals with tinnitus had an indirect effect on the third-party disability noticed by significant others (SOs). Methods: Significant Others Questionnaire (CTSOQ). Individuals with tinnitus completed standardized self-reported outcome measures for tinnitus severity, anxiety, depression, insomnia, hearing-related quality of life, tinnitus cognitions, hearing disability, and hyperacusis. Results: In total, 194 pairs of individuals with tinnitus and their SOs participated. The impact of third-party disability experienced by SOs was significantly reduced after individuals with tinnitus undertook the ICBT intervention (*d* = 0.41). This reduced SOs with severe difficulties from 52% to 35%. The remaining impact was mild for 30% and moderate for 35%. SOs with higher baseline difficulties and SOs who were partners (e.g., spouses) were less likely to notice indirect benefits from intervention undertaken by their family members. There was a moderate positive correlation between the post-intervention CTSOQs and the clinical variables of tinnitus severity and depression. Conclusions: Third-party disability may be reduced as an indirect effect of individuals with tinnitus undertaking ICBT. Including SOs of individuals with tinnitus within the rehabilitation process may add additional benefits, and such involvement should be encouraged.

## 1. Introduction

Traditional healthcare focuses on examining and treating only the presenting condition with the goal of resolving the underlying aetiology [1]. More recently, the interactions between organ systems and extrinsic modulating factors, including the influence of the environment and community, are increasingly recognised [2]. Healthcare models have thus shifted from considering individual health conditions in isolation to a more holistic approach, defined as consideration of the complete person, physiologically, psychologically, socially, and spiritually [3]. The impact of social and family support is also now increasingly acknowledged [4,5,6]. These complex interactions need to be particularly considered for conditions and symptoms that are difficult to diagnose, manage, and cure, such as tinnitus.

Tinnitus is one of the most frequently occurring chronic conditions in adults, with a point prevalence of 10–15% in adults [7,8]. Tinnitus is defined as the perception of sounds in an individual’s ears or head without any external sound source. Great variability is found in how those with tinnitus react to the tinnitus and respond to tinnitus treatments, with not all individuals showing improvements or finding the same type of intervention helpful. This may partly be related to the heterogeneity found regarding tinnitus variability [9]. There is, for instance, a bi-directional relationship between experiencing bothersome tinnitus as well as stress, anxiety, and depression [10,11,12]. Those severely affected by tinnitus may change many aspects of their daily activities to reduce exposure to sounds they think may aggravate the tinnitus. Some may thus reduce participation in household tasks, social gatherings, or activities in fear of negatively affecting the tinnitus [13,14]. The difficulties associated with tinnitus, together with the lifestyle changes, may thus have a direct impact on the significant others (SOs) of those with tinnitus, including relational difficulties, increased stress, and responsibilities. The impact of disability on not only the individual with the condition but also on those close to them—known as third-party disability—has been recognized in the World Health Organization’s International Classification of Functioning, Disability, and Health (ICF) framework [15]. Third-party disability is the consequence of a person’s disability, which impacts the functioning and ability of their SOs. SOs can be anyone with a close relationship with the individual with the disability, such as their spouse or another family member. Although this relationship is often between a partner and spouse, it may also be between other relatives or close friends.

Third-party disability has been recognized for many communication disorders, including hearing loss and balance disorders [16,17,18,19,20,21,22,23]. However, only a few studies have investigated the effect of third-party disability on tinnitus. Most studies examined the role of the spouse in mediating tinnitus experiences. A more recent qualitative study identified that tinnitus resulted in a reduction in SOs attending social events, music concerts, and functions due to individuals with tinnitus avoiding these situations [24]. SOs also reported an increased responsibility for household duties and childcare. In some cases, this has had an emotional toll due to the increased stress and frustration experienced by SOs, which in turn negatively affected the relationship between SOs and their individuals with tinnitus [24].

In an attempt to quantify third-party disability, Beukes et al. [25] developed and validated the Consequences of Tinnitus on Significant Others Questionnaire (CTSOQ). When using this structured questionnaire, it was found that tinnitus had a severe impact on 52%, a significant impact on 29%, and a mild impact on 19% of SOs with individuals with tinnitus [26]. Clearly, ways of addressing this disability are required. What is not known is whether reducing tinnitus distress in individuals with tinnitus will also indirectly reduce third-party disability in their SOs. Tinnitus distress is often reduced when managed by the provision of some sort of tinnitus intervention to help them better manage their tinnitus. Although there are many interventions, the one with the most evidence of effectiveness is cognitive behavioural therapy (CBT) for tinnitus [27,28]. CBT has also been developed into an online version (Internet-based cognitive behavioral therapy; ICBT) to increase access and decrease the resources associated with intervention delivery [29] and has shown to be an effective approach in reducing tinnitus and some of the associated difficulties (for reviews see [30,31]). It is possible that these improvements in the individual with tinnitus may also reduce third-party disability for their SOs, but this has not previously been established. The primary aim of the study was to investigate whether third-party disability experienced by SOs indirectly changed after individuals with tinnitus undertook ICBT. The secondary aims included (a) examining differences in characteristics of SOs who completed the post-intervention and those who did not; (b) identifying if there were any predictors that may identify which SOs would have a greater change in third-party disability following ICBT intervention for individuals with tinnitus; and (c) to examine if post-intervention outcomes between individuals with tinnitus and their SOs are related.

## 2. Materials and Methods

### 2.1. Recruitment

Individuals with bothersome tinnitus were invited to undertake 8 weeks of ICBT for tinnitus in English with the aim of reducing the problems associated with tinnitus [32,33,34]. Recruitment strategies target individuals with tinnitus and include a range of strategies, such as social media, flyers, e-mails, forums, and newsletters—which were distributed to local communities and put up in clinic waiting rooms. Professionals such as audiologists and otolaryngologists were also notified about the study. Those interested were directed to the study website, where they could read more about the study and register interest in partaking in the study. During registration, they had the option of selecting a significant other to join the study and pass the CTSOQ questionnaire link.

### 2.2. Participants

Participants consisted of pairs of individuals living in the USA, those with tinnitus, and their self-selected SOs. Study eligibility was determined as follows:

Inclusion criteria for individuals with tinnitus:

- Adults aged 18 years and over who experience tinnitus for a minimum period of 3 months. There was no maximum tinnitus duration.
- A tinnitus severity score of 25 or greater on the Tinnitus Functional Index (TFI) that indicates the need for an intervention.
- Any configuration of hearing levels (normal or any degree of hearing loss) and any use of hearing devices (using or not using hearing aids). Participants with hearing loss were contacted to ensure they were undertaking additional interventions for their hearing loss.
- Participants were to have access to a computer and the internet and not be undergoing any concurrent tinnitus therapies.
- Any type of tinnitus was considered. Participants with tinnitus that could be associated with other medical conditions, e.g., pulsatile or unilateral tinnitus, were contacted to ensure they were having investigations for this by medical professionals.

The exclusion criteria for individuals with tinnitus were:

- Indications of significant depression (≥15 scores) on the Patient Health Questionnaire PHQ-9
- Indications of self-harm thoughts or intent (i.e., answering affirming on Question 10 of the PHQ-9 questionnaire)
- Reporting any major medical, psychiatric, or mental disorder that may hamper commitment to the program or tinnitus as a consequence of a medical disorder still under investigation
- A clear protocol was set up for these patients. For any participant indicating possible self-harm thoughts or significant depression on the PHQ-9, a psychologist would phone them within 24 h for appropriate management.

Inclusion criteria for the significant others:

- SOs could be a spouse, partner, parent, adult child, sibling, other family members, housemate, or close friend who had a close emotional connection with the individual with tinnitus.

Both individuals with tinnitus and their SOs had to provide informed consent before completing the questionnaires.

### 2.3. Intervention

The ICBT intervention content was based on a Swedish CBT self-help program [35], transformed into an 8-week interactive e-learning version [36], and then adapted linguistically, culturally, and functionally to ensure suitability for a US population [37,38]. The ICBT platform consisted of 22 modules with worksheets and quizzes (see Beukes et al., [39] for more details). Participants were asked to read the modules weekly and ideally spend 10 min each day practicing the suggested strategies. Individuals with tinnitus were encouraged to share what they were learning during the course of the intervention, but the intervention was not designed for SOs. Guidance was provided by a trained therapist via an encrypted two-way messaging system to support participants while undertaking the intervention. This included monitoring progress, monitoring weekly scores, providing feedback on worksheets completed, outlining the content of new modules, and answering questions. The intervention specifically targeted reducing activity limitations and participation restrictions through behavioural change [40].

### 2.4. Data Collection

All the data were collected using online questionnaires. The questionnaire links were sent to the individual with tinnitus to complete. They were also asked to pass on the link to their SOs. The study thus relied on individuals with tinnitus to pass on the questionnaires to their SOs. Demographic information from all participants, such as age, gender, the relation between the SO and the individual with tinnitus, and whether they live with the individual with tinnitus, were collected at baseline. Those with tinnitus were asked who they had told about their tinnitus and if these people had helped in any way. Outcome measures were completed before undertaking the intervention (i.e., pre-intervention) and after undertaking the intervention for individuals with tinnitus (i.e., post-intervention).

### 2.5. Outcome Measures for Individuals with Tinnitus

Individuals with tinnitus completed a series of standardized outcome measures as follows: tinnitus severity measured by the Tinnitus Functional Index (TFI; [41]); anxiety measured by the Generalized Anxiety Disorder-7 (GAD-7; [42]); depression measured by the Patient Health Questionnaire-9 (PHQ-9; [43]); insomnia measured by the Insomnia Severity Index (ISI; [44]); tinnitus cognition measured using the Tinnitus Cognitions Questionnaire (TCQ; [45]); hearing disability and sound tolerance measured using the Tinnitus and Hearing Survey (THS; [46]).

### 2.6. Outcome Measures for Significant Others

SOs were asked to complete the CTSOQ, a custom-developed structured questionnaire focusing on the impact of tinnitus (i.e., third-party disability). Psychometric validation indicated good internal consistency (Cronbach’s α 0.93) and construct validity [25]. This structured questionnaire consists of 25 questions that focus on four sub-scales: (a) observations about the individual with tinnitus (10 questions); (b) personal impact (4 questions); (c) relationship impact (5 questions); and (d) providing support (6 questions). Each item is scored on a 5-point Likert scale (0 = strongly disagree, 1 = disagree, 2 = sometimes, 3 = agree, and 4 = strongly agree). The scores are added to range between 0 to 100, with higher scores indicating substantial effects of tinnitus on SOs and their relationship. Scores between 0–25 indicate a mild impact, scores between 26–40 a moderate impact, and scores of 41–100 a significant impact [25].

### 2.7. Data Analysis

Statistical analyses were performed using the Statistical Package for Social Sciences (SPSS) version 26.0 (IMB, 2019). All statistical tests were two-tailed with an alpha set to 0.05. Descriptive statistics, including age, gender, and the relationship between the SO and the individual with tinnitus, were used to describe the sample characteristics for each group. Continuous variables were summarized with means and standard deviations. Categorical variables were described using frequencies and percentages. Chi-square testing (for categorical variables) and a paired-sample *t*-test (for continuous variables) were used to identify any group differences regarding baseline characteristics between those with different CTSOQ scores and those completing and not completing the post-intervention assessment and those with and without tinnitus.

To identify if there were significant differences between pre and post-intervention scores, paired samples *t*-tests were used together with Cohen’s *d* (effect size testing). Effect sizes of *d* = 0.20 represent small effect sizes, those of *d* = 0.50 medium effect sizes, and those equal to or greater than *d* = 0.80 represent large effect sizes [47].

To identify if the CTSOQ score could be predicted from the clinical presentation of the individual with tinnitus, initially, correlations between the CTSOQ score and each clinical variable were explored. Pearson’s product–moment correlation coefficients were used to estimate the strength of the association between tinnitus severity and each variable. Correlation strength was categorized as very weak (0.00 to 0.19), weak (0.20 to 0.39), moderate (0.40 to 0.59), strong (0.60 to 0.79), and very strong (0.80 to 1.0). Following this, hierarchical multiple linear regression models were performed with the impact of tinnitus on SOs (i.e., CTSOQ scores) as the dependent variable and the tinnitus-related clinical variables as predictor variables. The data met the assumptions of homogeneity of variance, and the residuals were approximately normally distributed. There was no risk of multicollinearity, as indicated by the tolerances above 0.2 and variance inflation factor values below 10.

## 3. Results

### 3.1. Participants Characteristics

There were 194 eligible pairs of participants (individuals with tinnitus and their SOs) who completed the baseline questionnaires. The post-intervention questionnaire was returned by 148 (76%) of these individuals with tinnitus and 63 (32%) of the SOs. The age range was similar, with a mean of 55 (SD: 14) years for the SOs and 56 years (SD: 12 years) for the individuals with tinnitus. There were slightly more females (*n* = 117; 60%) compared with males (77; 40%) in the group with tinnitus, whereas 48% of the SOs were female (*n* = 94) and 52% were male (*n* = 100). The majority of the selected SO were partners (84%) and were living together with a person with tinnitus (87%), and only 18% had tinnitus themselves (Table 1). Although the ICBT intervention was intended for individuals with tinnitus, 10 (16%) of the SOs said that they had viewed the intervention materials, and 3 (5%) SOs mentioned trying the techniques.

### 3.2. Comparison of Significant Other Completing and Not Completing the Post-Intervention Questionnaire

The return rate for the SO questionnaire at post-intervention was low (32%). To identify if there were factors contributing to this, the characteristics of SOs, those completing and those not completing the post-intervention questionnaire, were compared (see Table 1). No difference between the groups was identified except that those completing the post-intervention questionnaire were more likely to have tinnitus themselves (21%) compared with those only completing the pre-questionnaire (9%).

### 3.3. Comparison of Significant Other with and without Tinnitus

Within- and between-group comparisons of SO with (*n* = 34; 18%) and without tinnitus (n = 160, 82%) are shown in Table 2. There were no significant differences in the CTSOQ pre-intervention or post-intervention scores for those SO with and without tinnitus. There were no differences in clinical outcomes for individuals with tinnitus when comparing whether the SO had tinnitus or not. Within-group comparisons indicated similar clinical outcomes for both SO and individuals with tinnitus regardless of whether the SO had tinnitus or not.

### 3.4. Impact of Tinnitus on the Significant Others

A reduction in scores on the CTSOQ was seen post-intervention. There was a significant difference of 5.59 (SD: 13.83) [*t*(622), *p* = 0.002] in the CTSOQ scores when comparing scores at baseline (mean: 40.92; SD: 17.32) and post-intervention (33.83, SD: 16.32). This indicated a moderate effect size of *d* = 0.41 (CI: 0.12 to 0.70).

**Figure 1:**
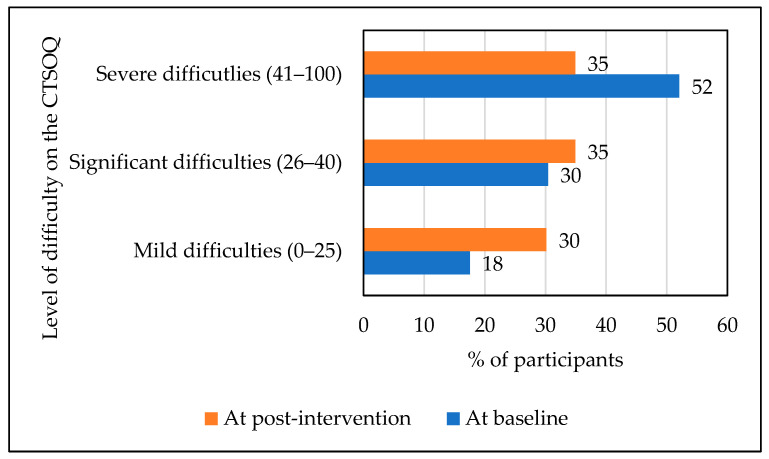
Score distribution regarding the impact of tinnitus on significant others using the Consequences of Tinnitus on Significant Others.

The distribution of scores is shown in Figure 1, indicating that 52% of SOs (*n* = 101) had severe difficulties at baseline when compared with 35% of SOs (*n* = 22) at post-intervention. Also, 30% of SOs (*n* = 19) had mild difficulties post-intervention when compared with 18% of SOs (*n* = 34) with mild difficulties at baseline.

### 3.5. Predictions of SO Outcomes at Post-Intervention

When investigating predictors of SOs post-intervention outcomes, a moderate positive correlation between the baseline and post-intervention CTSOQ score (*r* = 0.42, *p* = 0.04) was identified. A relationship regarding the post-intervention CTSOQ score and the kind of SO relationship was found (*χ*^2^ = 157, *p* = 0.03), as partners were more likely to have higher CTSOQ scores (mean: 34, SD: 17), and children with the lowest scores (mean: 28, SD: 12).

There was no correlation between the post-intervention score and the SOs’ age (*r* = 0.06, *p* = 0.63), gender (*χ*^2^ = 38.2, *p* = 0.64), living with the person with tinnitus (*χ*^2^ = 44.4, *p* = 0.37) or the SO themselves having tinnitus (*χ*^2^ = 51.5, *p* = 0.15).

### 3.6. Impact of Tinnitus on Individuals with Tinnitus

Individuals with tinnitus were asked who they had told about their tinnitus. The majority, 83% *(n* = 160), had told their partner, 71% (*n* = 138) told a good friend, 62% (*n* = 121) their children and/or parents and 28% (*n* = 55) an acquaintance. These SOs were not able to help in any way, according to 85% (*n* = 164) of individuals with tinnitus, although a small percentage (i.e., 15%; *n* = 30) reported their SOs had helped them with their tinnitus.

Comparison of the outcomes for individuals with tinnitus at baseline (i.e., pre-intervention) and post-intervention are shown in Table 2. Those with tinnitus indicated they had significant tinnitus distress with a score of 55 out of 100 on the TFI. Post-intervention, large effects were found for reducing tinnitus distress, insomnia, and negative tinnitus cognitions and moderate for reducing anxiety, depression, and hearing disability (Table 3).

### 3.7. Associations between the Significant Others Third-Party Disability and Post-Intervention Outcomes for Individuals with Tinnitus

There was a moderate positive correlation between the post-intervention CTSOQs and the clinical variables of tinnitus severity, depression, and insomnia (see the final column in Table 2). There was a weak positive relationship between the consequences of tinnitus on SOs and the clinical variables anxiety, tinnitus cognitions, and sound tolerance. There was no correlation between CTSOQ scores and hearing disability.

All significant variables were included in a multiple regression model. The hierarchical linear multiple regression model indicated that the clinical variables post-intervention from the individuals with tinnitus were able to predict the CTSOQ score of the SOs at post-intervention [*F*(8, 53) = 3.22, *p* = 0.005] and explained 57% of the variability of the CTSOQ score, although no variables were significant within the model due to the variables being highly correlated.

## 4. Discussion

Evidence is emerging regarding bothersome tinnitus, resulting in third-party disability to their SOs [24,26]. Ways of reducing this disability should be sought. The indirect effects of undergoing ICBT for tinnitus on SOs are unknown and were thus investigated in this study.

### 4.1. Indirect Effect of ICBT for Tinnitus on Significant Others

There was a significant reduction in CTSOQ scores for SOs at post-intervention, indicating a medium indirect effect (*d* = 0.41). The number of SOs experiencing severe difficulties was reduced from 52% at baseline to 35% at post-intervention. Although there were more SO with mild difficulties post-intervention (18%) than at baseline (30%), this proportion was relatively low compared with the numbers having significant and severe difficulties. During this study, the intervention was intended only for individuals with tinnitus and did not target SO at all. Nevertheless, the study demonstrated the indirect effect of ICBT in reducing third-party disability experienced by SOs of individuals with tinnitus. However, it may be important during future intervention development to consider how to further reduce third-party disability for SOs. Involving SOs in the intervention process may be one way. ICBT lends itself to this approach as individuals with tinnitus and their SO can jointly watch the intervention videos explaining more about tinnitus and strategies that may be helpful. They can also jointly apply suggested strategies, such as relaxation techniques, as these may be beneficial to both the person with tinnitus and their SO.

Research has indicated that tinnitus can impact the relationship between SO and individuals with tinnitus [24]; therefore, jointly undertaking ICBT may also have a positive effect on their relationship. Moreover, some specific content needs to be developed for SOs that may teach them techniques on how to deal with negative emotions and relationship issues, as well as ways to support individuals with tinnitus. Although no such intervention exists for SOs of individuals with tinnitus, there are examples of Internet-based CBT for SOs in other areas, such as gambling [48,49] and substance abuse [50].

### 4.2. Predictors of Outcomes Regarding Significant Others Characteristics

More difficulty was found post-intervention for those SOs with higher baseline scores on the CTSOQ. Greater difficulties were also found for those who were partners, such as spouses, compared with other relationships, e.g., children, parents, and friends. Thus, additional help should be sought for SOs showing higher baseline scores, and they should be encouraged to be involved in the intervention process, as learning more about tinnitus may be helpful. Spouses, in particular, should be encouraged to be involved. The impact of the closeness of the relationship may be a confounding variable. It has previously been identified that poor marital cohesion was significantly associated with greater tinnitus severity, anxiety, depression, and mediated maladaptive coping [51,52]. The reverse may also be applicable, that poor relationships may contribute to poorer post-intervention outcomes for both those with tinnitus and their SOs. When investigating third-party disability for hearing loss, it was found that lower relationship satisfaction contributed to third-party disability [53]. Tinnitus assessments and interventions should increase focus on a holistic approach and consider the impact of social and family support [3,4,5,6]. Where this support is lacking, including input from other support networks, such as buddy systems and tinnitus support networks, may be important [54]. No significant differences were found between the impact of tinnitus on SO with or without tinnitus, but this may be related to the assessment methods selected. Other measures and larger sample sizes may be required to explore this relationship further. Furthermore, although SOs were encouraged to explore the intervention materials, more joint exploration of the intervention materials should be encouraged, as well as ways to conduct this. This addition may be beneficial and highlight changes for SO with tinnitus and improve outcomes for individuals with tinnitus in future studies.

### 4.3. Association between Significant Others and Individuals with Tinnitus Post-Intervention Outcomes

It was found that post-intervention outcomes from persons with tinnitus could predict third-party disability of SOs post-intervention. For individuals with tinnitus who have poorer post-intervention outcomes with remaining tinnitus distress, depression, and insomnia, consideration should be given to the impact of these difficulties on their SOs. Recognising this interaction between difficulties experienced by individuals with tinnitus and their SOs is important, as SOs with greater difficulties may find it difficult to provide sufficient support, which can amplify problems for individuals with tinnitus. This, in turn, may negatively impact the relationship, which can again add to the tinnitus distress experienced.

### 4.4. Study Limitations and Future Directions

This is the first study to demonstrate the effect of ICBT for tinnitus on SOs, although it has a few limitations. This study has relied on individuals with tinnitus to pass on the questionnaires to their SOs. It was difficult to track whether the questionnaire was passed on or whether SOs selected not to complete the questionnaire. This may be related to the limited recommendations for who the significant other should be. Future research should specify how close significant others should be and how much concern and observation of the tinnitus they should have.

As completion rates for SOs were low post-intervention, it may be that the questionnaire was never passed on. It is difficult to track whether this was the reason or whether SOs selected not to complete the questionnaire. Future studies should directly involve SOs to overcome this barrier and identify reasons for poor completion. The study participants included individuals who were seeking online psychological interventions as a part of clinical trials [32,33,34] and their SOs. They had higher tinnitus severity, which may have resulted in higher third-party disability than SOs of typical tinnitus patients. For this reason, the current study sample may not be generalizable to the general tinnitus population, which is not as bothered by their tinnitus. Nevertheless, the study sample may be appropriate in the context of this study as more careful attention is needed on those who experience higher third-party disability. It would be helpful to identify the effect of third-party disability on a more general tinnitus population where not everyone with tinnitus finds it bothersome. Outcome measures evaluating wider effects, such as the impact on the relationship, the relationship assessment scale, and anxiety and depression measures for SOs, should be included in future studies. Also, no control group was added to this study. Future studies investigating whether third-party disability decreases over time without an intervention are required to identify the contribution of the intervention to this effect.

## 5. Conclusions

The current study is the first to identify an indirect benefit of ICBT in reducing the third-part disability on SOs of individuals with tinnitus. The study also showed that the benefits of ICBT may be less for SOs with greater third-party disability and SOs who are spouses or partners. These results suggest that these SOs may require intervention in their own right to deal with the negative consequences they experience as a result of their family member. These findings are important for both professionals involved with tinnitus interventions and policymakers who are considering healthcare priorities. Research should be mobilised to initiate joint intervention models for both individuals with tinnitus and their SOs.

## Figures and Tables

**Table 1:** Demographic profile of the significant others.

Demographics N (%)	SOs Completing the CTSOQ (*n* = 194)	SOs Completing the CTSOQ (*n* = 63)	SOs NOT Completing the CTSOQ (*n* = 131)	Difference between the SOs Completing and Not Completing the CTSOQ
Mean age in years ± Standard deviation	55 ± 14	55 ± 15	55 ± 13	*t*(122.388) = −0.138, *p* = 0.89
[range]	[18–84]	[19–82]	[18–84]	
Gender				*X*^2^(1) = 0.66, *p* = 0.72
Male	100 (52%)	34 (54%)	67 (51%)	
Female	94 (48%)	29 (46%)	64 (49%)	
Relationship				*X*^2^(4) = 5.53, *p* = 0.24
Partner	163 (84%)	51 (81%)	112 (85%)	
Parent	3 (2%)	0	3 (2%)	
Child	13 (7%)	7 (11%)	6 (5%)	
Relative	9 (4%)	4 (6%)	5 (4%)	
Friend	6 (3%)	1 (2%)	5 (4%)	
Living together *n* (%)				*X*^2^(1) = 0.42, *p* = 0.52
Yes	168 (87%)	56 (89%)	112 (86%)	
No	26 (13%)	7 (11%)	19 (14%)	
Presence of tinnitus				*X*^2^(1) = 4.13, *p* = 0.04
Yes	34 (18%)	6 (9%)	28 (21%)	
No	160 (82%)	57 (91%)	103 (79%)	
CTSOQ pre-intervention				*t*(149.875) = −0.967, *p* = 0.34
Mean score ± Standard deviation	40.92 ± 17.32	39.32 ± 14.75	41.69 ± 18.44	
[range]	[3 to 82]	[11 to 76]	[2 to 82]	
CTSOQ post-intervention			Not completed	NA
Mean score ± Standard deviation	33.83 ± 16.32	33.83 ± 16.32		
[range]	[2 to 69]	[2 to 69]		

Acronyms: SO: significant others; CTSOQ: Consequences of Tinnitus on Significant Others Questionnaire.

**Table 2:** Within- and between-group comparisons for significant others with and without tinnitus.

	Within Group Comparisons		Between Group Comparisons
Clinical Variables	SOs with Tinnitus (*n* = 24, 18%)	SOs without Tinnitus (*n* = 160, 82%)	Group Differences between SO with and without Tinnitus
CTSOQ ^1^ pre-intervention	*t*(33) = 13.00, *p* =< 0.001 *d* = 2.23 [1.59 to 2.86]	*t*(159) = 30.17, *p* =< 0.001 *d* = 2.38 [2.08 to 2.69]	*X*^2^(63) = 63.07, *p* = 0.47
CTSOQ post-intervention	*t*(5) = 3.57, *p* = 0.008 *d* = 1.46 [0.24 to 2.61]	*t*(56) = 16.19, *p* =< 0.001 *d* = 2.16 [1.65 to 2.58]	*X*^2^(42) = 51.40, *p* = 0.15
TFI	*t*(24) = 7.13, *p* =< 0.001 *d* = 1.38 [0.83 to 1.98]	*t*(159) = 15.40, *p* =< 0.001 *d* = 1.36 [1.12 to 1.60]	*X*^2^(100) = 113.80, *p* = 0.16
GAD-7	*t*(24) = 5.26, *p* =< 0.001 *d* = 1.02 [0.54 to 1.49]	*t*(125) = 11.47, *p* =< 0.001 *d* = 1.02 [0.81 to 1.24]	*X*^2^(16) = 5.67, *p* = 0.99
PHQ-9	*t*(24) = 4.38, *p* =< 0.001 *d* = 0.86 [0.40 to 1.32]	*t*(125) = 10.74, *p* =< 0.001 *d* = 0.96 [0.74 to 1.17]	*X*^2^(17) = 16.80, *p* = 0.57
ISI	*t*(24) = 7.22, *p* =< 0.001 *d* = 1.43 [0.85 to 1.98]	*t*(124) = 13.76, *p* =< 0.001 *d* = 1.22 [0.99 to 1.45]	*X*^2^(22) = 29.38, *p* = 0.13

^1^ Acronyms: SO: significant others; CTSOQ: Consequences of Tinnitus on Significant Others Questionnaire TFI = Tinnitus Functional Index; GAD-7 = Generalised Anxiety Disorder; PHQ-9 = Patient Health Questionnaire; ISI = Insomnia Severity Index.

**Table 3:** Outcome measures of the individuals with tinnitus at baseline and post-intervention, as well as a correlation between post-intervention scores for individuals with tinnitus and third-part disability experienced by significant others.

Clinical Variables	Individuals with Tinnitus at Baseline (*n* = 194)	Post-Intervention Score (*n* = 148)	Effect Size at Post-Intervention	Correlation with the Post-Intervention Score from the Consequences of Tinnitus on Significant Others Questionnaire
	Mean ± Standard Deviation [range]	Mean ± Standard Deviation [range]	Cohen’s *d* [Confidence Interval]	Pearson’s Correlation
		Mean (Standard Deviation) [range]	Cohen’s *d* [Confidence Interval]	Pearson’s Correlation
TFI	55.01 ± 20.32 [7–96]	29.56 ± 21.45 [0–100]	*d* = 1.22 [0.99 to 1.46]	*r* = 0.46, *p* < 0.001
GAD-7	7.11 ± 5.29 [0–21]	4.17 ± 4.08 [0–21]	*d* = 0.61 [0.39 to 0.83]	*r* = 0.37, *p* = 0.003
PHQ-9	7.23 ± 5.47 [0–26]	4.21 ± 4.44 [0–27]	*d* = 0.60 [0.38 to 0.82]	*r* = 0.43, *p* < 0.001
ISI	11.3 ± 6.24 [0–27]	6.96 ± 5.50 [0–28]	*d* = 0.73 [0.51 to 0.95]	*r* = 0.43, *p* < 0.001
TCQ	43.14 ± 16.16 [2–89]	29.20 ± 17.01 [0–100]	*d* = 0.84 [0.62 to 1.77]	*r* = 0.28, *p* = 0.03
THS	6.81 ± 4.55 [0–16]	4.60 ± 3.70 [0–16]	*d* = 0.52 [0.31 to 0.74]	*r* = 0.23, *p* = 0.08
THS	1.13 ± 1.31 [0–4]	0.77 ± 1.05 [0–4]	*d* = 0.30 [0.08 to 0.51]	*r* = 0.39, *p* = 0.002

Accroynms: TFI = Tinnitus Functional Index; GAD-7 = Generalised Anxiety Disorder; PHQ-9 = Patient Health Questionnaire; ISI = Insomnia Severity Index; TCQ = Tinnitus Cognitions Questionnaire; THS = Tinnitus and Hearing Survey.

## Data Availability

Data are available from http://doi.org/10.6084/m9.figshare.15062691.
